# Hairy root induction and benzylisoquinoline alkaloid production in *Macleaya cordata*

**DOI:** 10.1038/s41598-018-30560-0

**Published:** 2018-08-10

**Authors:** Peng Huang, Liqiong Xia, Wei Liu, Ruolan Jiang, Xiubin Liu, Qi Tang, Min Xu, Linlan Yu, Zhaoshan Tang, Jianguo Zeng

**Affiliations:** 1 Hunan Key Laboratory of Traditional Chinese Veterinary Medicine, Hunan Agricultural University, Changsha, Hunan 410128 China; 2 College of Horticulture and Landscape, Hunan Agricultural University, Changsha, Hunan 410128 China; 3 School of pharmacy, Hunan University of Chinese Medicine, Changsha, 410208 China; 4 Center of Analytic Service, Hunan Agriculture University, 410208 Changsha, China; 5 Micolta Bioresource Inc., Changsha, 410016 China; 6 National and Local Union Engineering Research Center of Veterinary Herbal Medicine Resource and Initiative, Hunan Agricultural University, Changsha, Hunan 410128 China

## Abstract

Sanguinarine is currently widely used to replace antibiotic growth promoters in animal feeding and has demonstrated useful anticancer activity. Currently, the main source of sanguinarine is from an important medicinal plant, *Macleaya cordata*. To obtain a new source of sanguinarine production, we established hairy root cultures of *M*. *cordata* by co-cultivating leaf and stem explants with *Agrobacterium rhizogenes*. Except the co-cultivation medium, all growth media contained 200 mg/L timentin to eliminate *A*. *rhizogenes*. Through comparing the metabolic profiles and gene expression of hairy roots and wild-type roots sampled at five time points, we found that the sanguinarine and dihydrosanguinarine contents of hairy roots were far higher than those of wild-type roots, and we revealed the molecular mechanism that causes these metabolites to increase. Consequently, this study demonstrated that the hairy root system has further potential for bioengineering and sustainable production of sanguinarine on a commercial scale. To the best of our knowledge, this is the first efficient protocol reported for the establishment of hairy root cultures in *M*. *cordata* using *A*. *rhizogenes*.

## Introduction

Sanguinarine (SAN) is a quaternary benzylisoquinoline alkaloid (BIA). SAN has been used for many years as a natural growth promoter (NGP) and alternative to antibiotics in livestock production^1,2^. Currently, the European Union and USA have banned the use of antibiotic growth promoters (AGPs) in livestock husbandry^3^. As a result, the consumer market for NGPs has rapidly expanded; the global consumption of SAN has increased steadily each year, and the market is expected to exceed 300 million euros. Recently, SAN has been shown to have potential uses in treating schistosomiasis and osteoarthritis^4–6^. In addition, this chemical has attracted the attention of many pharmacologists because of its multiple biological activities, such as antitumour^7–13^, antimicrobial^14^ and anti-inflammatory^1^. However, SAN is difficult to obtain by chemical synthesis because of its structural complexity. To date, wild or cultivated plants remain the only way to obtain SAN for commercial use. *Macleaya cordata* (Chinese name “Bo-luo-hui”) is a traditional medicinal herb that belongs to the Papaveraceae family. In addition, it is the most important commercial source of SAN^3,15,16^. This plant has been approved by the European Food Safety Authority (EFSA) as a safe plant for the manufacture of feed additives^16^. At present, the commercial supply of SAN comes mainly from the capsules of *M*. *cordata*, but field cultivation and collection are time-consuming and labour-intensive processes. Increasing labour costs will further raise the production price of SAN. Consequently, the need to develop a sustainable and commercially scalable production method for SAN through plant *in vitro* culture technology has become urgent.

Hairy roots (HR) have been used for metabolic engineering in the past^17^. Especially in recent years, HR cultures have become a useful biological system to study the biosynthesis of alkaloids^18,19^. In addition, HR display higher biochemical stability than that of other plant cultures. More importantly, HR often allow large-scale biomass and phytochemical production^20^. In recent years, various bioactive compounds, including tropane alkaloids and nicotine^21^, ginsenosides^22,23^, anthraquinones^24^ and *Artemisinin*^25^ have been produced by HR culture. Notably, bioreactor technology for mass cultivation of *Panax ginseng* and *Artemisia annua* has been established^20,25^. Thus, HR cultures of *M*. *cordata* provide a promising prospect for industrial-scale harvest of SAN resources.

**Figure 1:**
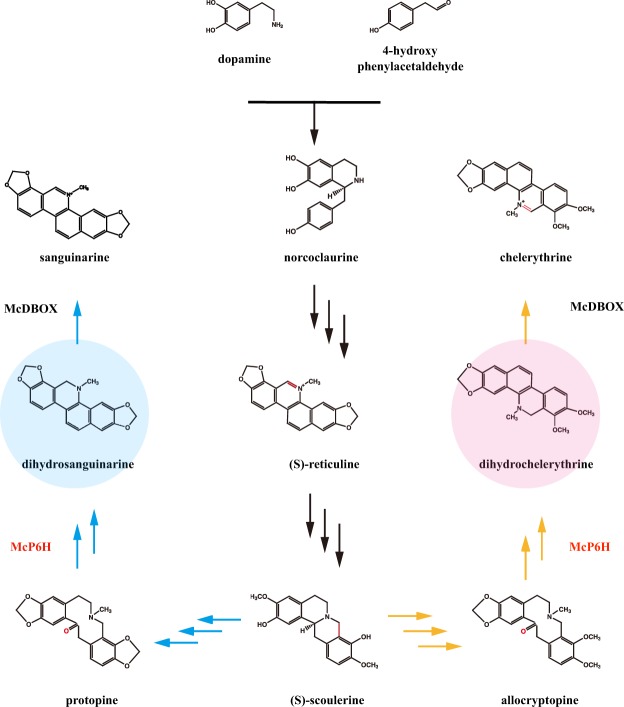
The metabolic pathway of sanguinarine and chelerythrine. Protopine 6-hydroxylase (McP6H) and dihydrobenzophenanthridine oxidase (McDBOX) were studied in this work.

To the best of our knowledge, no research exists about HR cultures of *M*. *cordata*. In this work, we established an HR culture system for *M*. *cordata*, evaluated different types of explants and developed a suitable protocol. Our recent work includes functional characterizations of many metabolic genes involved in SAN biosynthesis and identification of the pattern of SAN synthesis in *M*. *cordata*^16^. In another previous study, we identified the protopine 6-hydroxylase (McP6H) and dihydrobenzophenanthridine oxidase (McDBOX) enzymes involved in the conversion of protopine (PROT) to SAN in *M*. *cordata*. However, these two genes are also involved in the biosynthesis of chelerythrine (CHE), another active compound in *M*. *cordata*, along another branch of the biosynthetic pathway (Fig. 1). Therefore, in this study, we compared the metabolic profiles of HR and wild-type (WT) roots through ESI/QQQ MS analysis and detected the expression of McP6H and McDBOX at five time points. Finally, we confirmed the functionality of the transgenic system and the integration of *rol* genes by molecular biological analysis.

## Results

### Establishment of hairy root cultures

**Figure 2:**
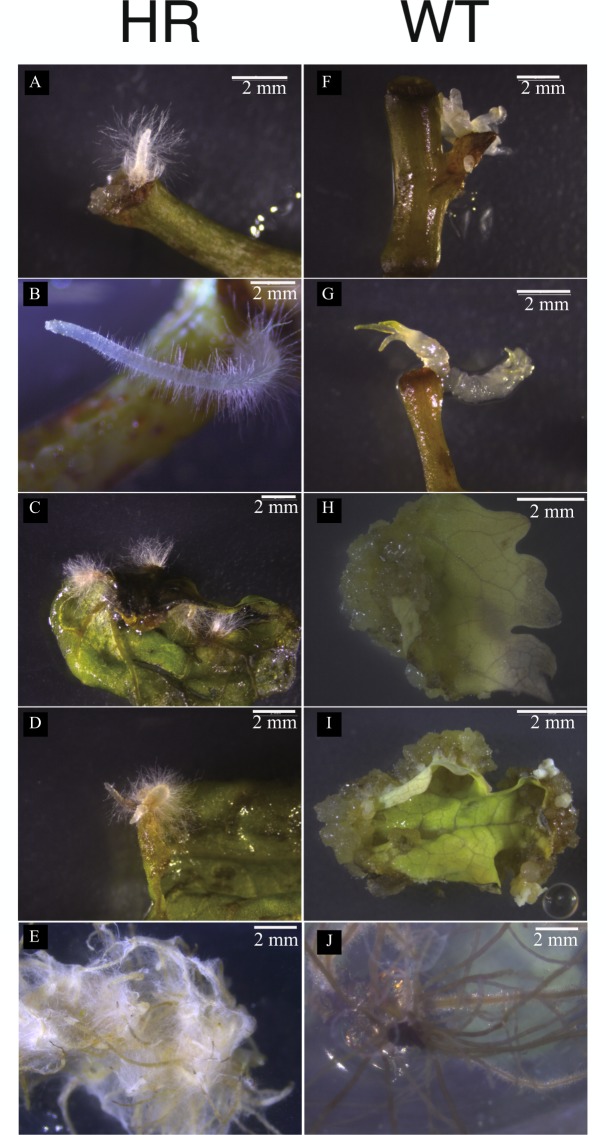
Induction of hairy roots from different explants of *M*. *cordata* compared with the wild type (**A**,**B**) Initiation of hairy roots on stem wounds after 15–30 days of infection. (**C**,**D**) Initiation of hairy roots on leaf wounds after 15–30 days of infection. (**E**) The well-established hairy root cultures. (**F**,**G**) Initiation of embryogenic calli on stem wounds after 15–30 days. (**H**,**I**) Initiation of embryogenic calli on leaf wounds after 15–30 days. (**J**) The wild type roots (shooting from the bottom of the bottle). Scale bars represent 2 mm.

The method we used for *A*. *rhizogenes* infection comes from an improvement on the previous transformation system of *M*. *cordata*^26^. In this study, an *A*. *rhizogenes* strain (10060) was used to induce two types of explants (leaves, stems) of *M*. *cordata* to form HR. Initials emerged from the wounded parts of the leaves and stems within 7–10 days after co-cultivation. After 15–20 days, HR of *M*. *cordata* began to grow more rapidly (Fig. 2A–D). After 5–6 weeks, the HR were isolated from the explants and subcultured on selection medium. Observations showed that the HR had a high rate of lateral branching and produced a greater abundance of HR than WT did. Interestingly, unlike WT explants that produce large amounts of callus prior to the formation of roots, HR can emerge directly from the wounds. After several successive subcultures, no bacteria were visible on the surface of the medium, indicating that the residual bacteria had been completely removed. All the explants could be induced by this strain to form HR (Fig. 2). The rates of induction in the leaves and stems were 4.11 ± 1.01% and 38.06 ± 3.84%, respectively (Table 1).

**Table 1:** Effects of different explants on hairy root induction rate.

Explants	Number of explants	Number of hairy roots	Induction rate (%)
Leaf	104.3 ± 4.2	4.3 ± 1.3	4.11 ± 1.01
Stem	113.3 ± 4.9	43.3 ± 6.3	38.06 ± 3.84^**^

Asterisks denote significant changes (Tukey’s test, P < 0.05) between the two groups. Data represent the means of triplicate cultures ± standard deviation.

### PCR amplification

**Figure 3:**
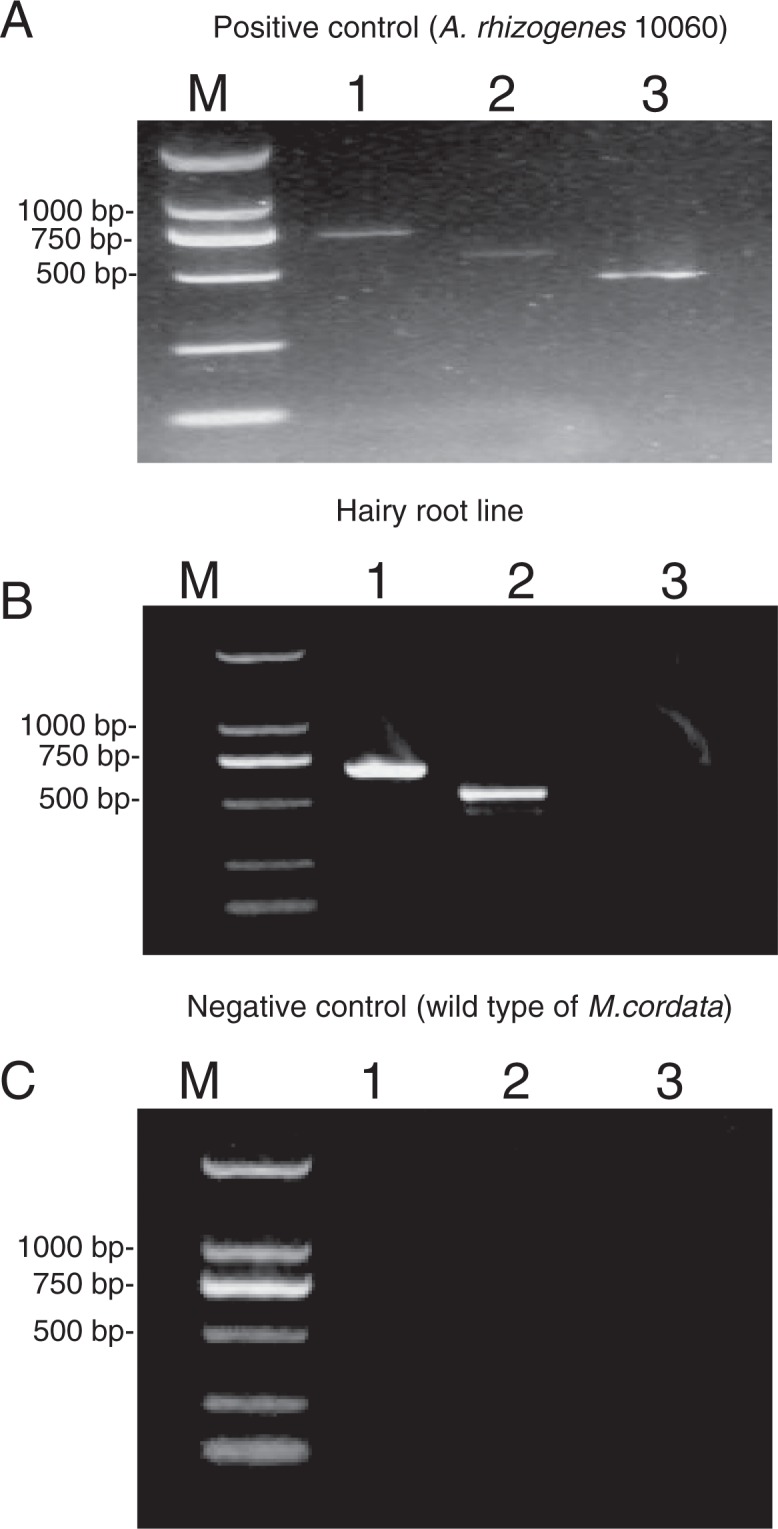
PCR analysis of (**A**) positive control (*A*. *rhizogenes* 10060), (**B**) *M*. *cordata* hairy root line, (**C**) negative control (wild-type root of *M*. *cordata*). The three lanes shown in (**A–C**), from left to right, represent the (1) rolB (670 bp), (2) rolC (534 bp) and (3) virD gene PCR products (438 bp).

In our study, the *A*. *rhizogenes* 10060, HR and WT were analysed by PCR. In addition, we used gene-specific primers for virD to exclude bacterial contamination. Figure 3 displays the PCR assay used to identify the *rol*B, *rol*C and VirD genes in three samples. The *rol*B, *rol*C and VirD genes were detected in the *A*. *rhizogenes* 10060. The PCR analysis of HR produced *rol*B and *rol*C but did not detect VirD. In addition, no product of PCR amplification was observed in WT.

### Alkaloid accumulation and expression of SAN and CHE biosynthetic genes in HR and WT

**Figure 4:**
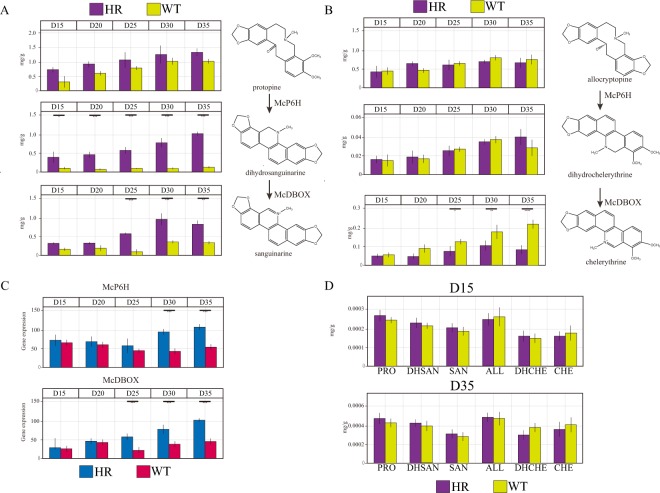
Time course analysis of alkaloid levels and gene expression in HR, WT and spent medium. (**A**) The time course analysis of the alkaloids in the SAN branch of the biosynthetic pathway (PROT, DHSAN, SAN) in HR and WT. (**B**) The time course analysis of the alkaloids in the CHE branch of the biosynthetic pathway (ALL, DHCHE, CHE) in HR and WT. (**C**) The time course analysis of McP6H and McDBOX gene expression in HR and WT. (**D**) The time course analysis of alkaloids in spent medium. Asterisks denote significant changes (Tukey’s test, P < 0.05) between two comparable groups.

To investigate the differences in BIA biosynthetic profiles between HR and WT, HR and WT samples were measured by LC-MS/MS and ABI 7300 at five time points. Then, we analysed the contents of 6 alkaloids (PROT, DHSAN, SAN, ALL, DHCHE, CHE) and the expression levels of SAN and CHE biosynthetic genes (McP6H, McDBOX) in HR and WT (Fig. 4A–C). Overall, the contents of the 3 alkaloids on the SAN branch of the biosynthetic pathway (PROT, DHSAN, SAN) in HR were significantly higher than those in WT, especially DHSAN and SAN (P < 0.05), and the content of DHSAN increased with the growth time (Fig. 4A). Notably, in 35-day tissues (35D), the contents of DHSAN and SAN were higher (11.3 and 2.3 times, respectively) than those of WT. However, the contents of the remaining three alkaloids (PROT, ALL, DHCHE) were similar in both groups (Fig. 4B). According to the qPCR results (Fig. 4C), the gene expression levels of McP6H and McDBOX in all HR lines were higher than those in all WT lines, and they showed significant increases in the tissues at 30–35 days (30–35D). Finally, we tested the contents of the 6 alkaloids in the solid medium at 15 and 35 days (15D, 35D) but found little difference between the two groups.

## Discussion

The induction of HR by *A*. *rhizogenes* has been widely used for the production of secondary metabolites and as a model efficient expression system in functional genomics. In the past few decades, many important medicinal plants have been established as HR cultures^27^, especially *Papaver somniferum* and *Eschscholzia californica*^18,28–30^. However, the conversion rate of *P*. *somniferum* hairy root was 80%, twice that of *M*. *cordata* (40%). In this study, *A*. *rhizogenes* strain 10060 was used in the induction of *M*. *cordata* HR, while LBA 9402 and R1000 were used in *P*. *somniferum and E*. *californica* HR, respectively^18,28^. This experiment proved that stem tissue is a suitable explant for HR induction in *M*. *cordata*, and we know that the stem tissue is more conveniently obtained than the hypocotyls of *P*. *somniferum*. Additionally, morphological observations found that the HR of *M*. *cordata* resemble the HR of *P*. *somniferum* (thin morphology), although the HR of *P*. *somniferum* were grown in LS liquid medium^28^, while the *M*. *cordata* HR were cultured on hormone-free solid medium. The HR of *M*. *cordata* produced more alkaloids than untransformed roots did, and only traces of alkaloids were detected in the spent medium. In our study, we used an efficient method for establishing HR in *M*. *cordata*. In the putative HR, we used PCR amplification to prove that the *rol* genes were integrated into the *M*. *cordata* genomic DNA. This result demonstrates that stable and integrative transformation of *M*. *cordata* HR cultures was achieved. To the best of our knowledge, this is the first report of *M*. *cordata* HR cultures producing BIAs. In previous research, the transgenic cell culture system of *M*. *cordata* was established using *A*. *tumefaciens*^26^. However, there are two reasons for us to construct an *A*. *rhizogenes*-mediated hairy root system. First, the life cycle is too long to efficiently obtain SAN from the seeds of *M*. *cordata*, requiring almost a full year to obtain the capsules of *M*. *cordata*, which are the main resource used to extract SAN; thus, HR can shorten the time to SAN production. Second, we believe that the roots of *M*. *cordata* are the main location of BIA biosynthesis. Therefore, we hope to increase the production of BIAs and obtain high-yield lines by establishing an HR system that stimulates BIA biosynthesis. We based the protocol on previous research: the explants were subjected to vacuum for 10 min, and the concentration of antibiotic was 200 mg/L timentin. In this study, leaves and stems from 2-month-old aseptic seedlings were used for inoculation. The results indicated that HR induction from leaves was too low (4.11 ± 1.01), and all wounded leaves and explants turned brown within 3 weeks. In contrast, the induction rate of stems was nearly 40%. Additionally, unlike in leaves, the HR emerged from stem callus within 10–15 days. Therefore, comparing the induction rates of the different explants revealed that the stem was the most suitable material to initiate HR.

Notably, the five-point time course showed that the contents of 3 alkaloids (PROT, DHSAN, SAN) were significantly higher in HR than in WT. In the metabolic results, we observed that 3 metabolites (PROT, DHSAN and SAN) were significantly increased (Fig. 4A), while the levels of other alkaloids in the CHE branch of the pathway (ALL, DHCHE, CHE) were not obviously altered (Fig. 4B). These differences can be explained by gene expression changes. The expression of the McP6H and McDBOX genes, which play key roles in SAN biosynthesis, increased significantly in HR. This result could be attributed to the influence of endogenous auxin synthesis controlled by the rol genes in the HR. The rol genes include rolA, rolB, and rolC, and previous research has indicated that rol B is probably the most powerful inducer of secondary metabolism^31^. Additionally, this study provided new insights into the substrate preferences of the McP6H and McDBOX enzymes. We observed that McP6H and McDBOX primarily enhanced the SAN branch of the biosynthetic pathway; this result might indicate the substrate preferences of both enzymes. Previous studies have indicated that the roots and capsules of mature *M*. *cordata* are the main parts that biosynthesise SAN and CHE. In addition, SAN and CHE are mainly stored in the capsules after synthesis during the mature period. In conjunction with these findings, we thought that the HR of *M*. *cordata* could both biosynthesise and store SAN because no other organs are present. Due to the large amount of DHSAN stored in the HR, we could overexpress the McDBOX gene in HR to further improve SAN production. Although the HR system has been successfully established, the current size of the HR is still much less than that of field roots. Therefore, in the next step, we will screen more suitable high-production lines using a variety of *A*. *rhizogenes* strains.

## Conclusion

This study is the first report of the application of HR culture in *M*. *cordata*. In the present investigation, we have successfully established an *M*. *cordata* HR system and compared the metabolic profiles of HR and WT. Using vacuum treatment, HR can be obtained within a relatively short time (5–7 weeks); this method is simple and convenient. The induction frequencies of HR in leaves and stems were 4.11 ± 1.01% and 38.06 ± 3.84%, respectively. Therefore, stems were the explants of choice for HR induction. The best conditions for transformation included the use of stem explants and coculture for a 3-day period in MS medium supplemented with 20 mg/L acetosyringone. Elevated levels of the metabolites on the SAN branch of the biosynthetic pathway were observed in all the HR lines. At the same time, the amounts of McP6H and McDBOX gene expression in HR were much greater than those in WT, which shows that the expression pattern of the HR material was unique. Although SAN is the most important compound in intact field-grown *M*. *cordata*, DHSAN is the immediate precursor to SAN. Therefore, this HR system provides a potential method to further increase SAN content. In conclusion, this study describes a method for HR induction, which could be useful in the future for bioengineering and for sustainable production of SAN on a commercial scale by HR culture of *M*. *cordata*.

## Materials and Methods

### Preparation of *A*. *rhizogenes* strain

The *A*. *rhizogenes* strain (10060) was purchased from BioRc Co. Ltd (China). The strain was streak-cultured on solid YEB medium (containing 10 g/L tryptone, 5 mg/L yeast extract and 10 mg/L NaCl, pH 7.2) and 100 mg/L of rifampin (Rif), and then incubated at 28 °C overnight. The next day, single colonies were inoculated into liquid YEB medium (100 mg/L Rif) and incubated at 28 °C on a shaker at 170 rpm overnight. Then, the bacteria were collected by centrifugation at 1057 × g for 15 min and re-suspended in MS liquid medium (containing 30 g/L sucrose and 20 mg/L acetosyringone) to OD ≈0.4 for infection.

Leaves and stems were chosen from 2-month-old sterile *M*. *cordata* plants for use as explants. All sterile plants were grown in our laboratory. The explants were submerged in the *A*. *rhizogenes* suspension medium and submitted to a continuous vacuum for 10 min^26^. Then, we put the explants on sterile filter paper to remove surface moisture and excess bacteria and incubated them in darkness on co-cultivation medium (MS solid medium, 30 g/L sucrose, 8 g/L agar, pH 5.8) at 25 °C for 3 days. In previous studies, we found that 20 mg/L acetosyringone in the co-cultivation medium was the most effective concentration for induction of transformants^26^. Therefore, we used this concentration in the co-cultivation medium. After 3 days, the explants were transferred to MS solid medium (hormone-free, 200 mg/L timentin). All explants were incubated at 25 ± 2 °C under 16 h light and 8 h dark. The cultures were transferred every 20 days to fresh solid medium with timentin. Adventitious roots were observed emerging from the wound sites of the explants by 6–10 days after co-cultivation. After 5–6 weeks, single roots could be isolated from the explants and cultured on fresh hormone-free MS solid medium (200 mg/L timentin) at 25 ± 1 °C in the dark and subcultured every 3 weeks. During this period, the concentration of timentin was gradually reduced and finally omitted, until the bacteria were eliminated completely.

### DNA extraction and PCR amplification

The genomic DNA of *M*. *cordata* was isolated from the putative HR lines and WT using the TIANGEN DNeasy Plant Mini Kit (TIANGEN, China). The Ri plasmid genes *rol*B (670 bp) and *rol*C (534 bp) and the plasmid virulence gene virD (438 bp) primers were used in PCR analysis to confirm the integration of the T-DNA during HR formation (Table 2). The plasmid from *A*. *rhizogenes* 10060 was used as the positive control, and WT was used as the negative control. The PCR amplification (TAKARA, China) program was as follows: 94 °C for 5 min, followed by 30 cycles of 94 °C for 1 min, 58 °C for 30 s, and 72 °C for 30 s and a final extension at 72 °C for 5 min. The amplification products were analysed by 1% (w/v) agarose gels prepared in 0.5× TBE (Tris/Borate/EDTA) buffer.

**Table 2:** Primers used for PCR analysis.

Name	Sequence
*rol*B-F	TAGCCGTGACTATAGCAAACCCCTCC
*rol*B-R	GGCTTCTTTCTTCAGGTTTACTGCAG
*rol*C-F	TAACATGGCTGAAGACGACC
*rol*C-R	AAACTTGCACTCGCCATGCC
virD-F	ATGTCGCAAGGCAGTAAGCCCA
virD-R	GGAGTCTTTCAGCAGGAGCAA

### Time course collection of spent medium and *M*. *cordata* plant tissues

After the HR were isolated from the explants and cultured on MS solid medium (200 mg/L timentin) at 25 ± 1 °C in the dark, we collected HR samples after 15, 20, 25, 30, and 35 days (15D, 20D, 25D, 30D, 35D) for testing. In addition, WT root samples were collected at the same times (15, 20, 25, 30, and 35 days) as controls. Since the medium was changed every 15 days, we chose the spent medium from HR and WT at 15, 25, and 35 days for testing.

### Metabolite extraction and LC-QQQ MS analysis

HR and WT tissues were collected after 30 days of culture, ground into a fine powder using liquid nitrogen and then freeze-dried. Then, ultrasonic extraction was performed for 30 min at room temperature in 1 mL of methanol, followed by ultrasonic extraction for 60 min at room temperature to isolate the metabolites from 50 mg of tissue. After filtration through a 0.22-mm membrane filter (Pall, USA), the solution was quantitatively analysed by LC/triple-quadrupole (QQQ) MS. An ultra-HPLC Agilent 1290 instrument coupled to a QQQ mass spectrometer (6460 A, Agilent) with a BEH C18 column (2.1 3 100 mm, 1.8 mm; Waters, Ireland) was used for the determination of 6 target alkaloids [protopine (NIFDC, China), dihydrosanguinarine (Micolta, China), sanguinarine (NIFDC), allocryptopine (Micolta, China), dihydrochelerythrine (Micolta, China), chelerythrine (NIFDC)]. The quantitative analysis of HR and WT metabolites was performed according to our previous research^16^. The operating parameters were as follows: the flow rate was 0.3 mL/min and the injection volume was 2 mL, nebulizer gas pressure, 55 psi; and capillary voltage, + 3500 V for ESI+. The quantitative ion pairs were 354.1 → 189.0, 370.2 → 188.0, 332.1 → 217.0, 348.1 → 304.0, 350.1 → 334.0 and 334.1 → 318.0 for PROT, allocryptopine (ALL), SAN, CHE, dihydrochelerythrine (DHCHE) and dihydrosanguinarine (DHSAN), respectively. Data acquisition was performed in multiple reaction modes. All alkaloids were detected in MRM mode. The LC-QQQ MS data were processed using the Agilent Mass Hunter Quantitative Analysis software (B.07.00). For absolute quantification analysis, the method was validated using a mixed standard solution, which was diluted with methanol to produce at least 5 points and was used to evaluate the absolute quantification of the target compound.

### Gene expression analysis by quantitative PCR

Total RNA was isolated from putative HR and WT of *M*. *cordata* using the MiniBEST Plant RNA Extraction Kit (TaKaRa, China). The quality of the RNA was checked by agarose gel electrophoresis, and its quantity was confirmed by Qubit 2.0. Subsequently, cDNA was synthesized from 0.5 mg of total RNA using a PrimeScript RT reagent Kit (TaKaRa, China). The resulting cDNA products were diluted to 100 μL for use as templates in subsequent experiments. PCR was performed on an ABI 7300 using Fast Start Universal SYBR Green Master (ROX) according to the manufacturer’s instructions. The total volume of the quantitative real-time PCR assay was 20 μL (10 μL of PCR Mix, 0.5 μL of specific primers, 4 μL of cDNA and 5 μL of water). The qPCR cycling protocol was as follows: 95 °C for 15 min; then, 40 cycles of 95 °C for 15 s, 55 °C for 15 s, and 72 °C for 20 s. In this method, three replicates and three independent biological experiments were performed in all cases. Relative gene expression was determined using the comparative 2^−△△Ct^ or 2^−△Ct^ methods. All the primer sequences were from^16^, and the 18S gene was used as the internal reference (Table 3).

**Table 3:** Primers used for qPCR analysis.

Name	Sequence
McP6H-F	CATCAAGGACGTTCGAGCCT
McP6H-R	CTCCTCACCACGCACAATCT
McDBOX -F	ACTGTTGCCACGGTCGATAG
McDBOX-R	TGGAGGAGCTTGTCAACACC
Mco18S-F	CTTCGGGATCGGAGTAATGA
Mco18S-R	GCGGAGTCCTAGAAGCAACA

### Statistical analysis

All the experiments including HR induction, quantitative PCR analysis, RT-PCR, and LC-QQQ MS analysis were repeated three times. All the results are presented as the mean values ± S.D. The differences between the means were determined by analysis of variance with Tukey’s test using GraphPad Prism statistical software (version 7.0, USA), and the level of significance was set at P < 0.05.
